# Prognostic Value of the C-Reactive Protein-to-Albumin Ratio in Patients Hospitalized with Atrioventricular Block: Association with In-Hospital Mortality and Temporary Pacemaker Requirement

**DOI:** 10.3390/medicina62101993

**Published:** 2026-10-15

**Authors:** Kemal Emrecan Parsova, Levent Pay, Ahmet Cagdas Yumurtas, Tugba Cetin, Feyza Mollaalioglu, Seyda Dereli, Mert Ilker Hayıroglu, Kadir Gurkan

**Affiliations:** 1 Department of Cardiology, Koc University Hospital, Istanbul 34010, Turkey; 2 Department of Cardiology, Basaksehir Cam & Sakura City Hospital, Istanbul 34480, Turkey; 3 Department of Cardiology, Foundation of the Cellitinnen to the Holy Maria, 50126 Bergheim, Germany; 4 Department of Cardiology, Corlu State Hospital, Tekirdag 59850, Turkey; 5 Department of Cardiology, Cerkezkoy State Hospital, Tekirdag 59500, Turkey; 6 Department of Cardiology, Golbası State Hospital, Adıyaman 02500, Turkey; 7 Department of Cardiology, Acibadem Atasehir Hospital, Istanbul 34642, Turkey; 8 Department of Cardiology, Dr. Siyami Ersek Thoracic and Cardiovascular Surgery Training and Research Hospital, University of Health Sciences, Istanbul 34668, Turkey

**Keywords:** atrioventricular block, C-reactive protein-to-albumin ratio, in-hospital mortality, inflammation, temporary pacemaker

## Abstract

*Background and Objectives*: The C-reactive protein-to-albumin ratio (CAR) is an available marker of inflammatory–nutritional vulnerability, but its prognostic value in atrioventricular (AV) block is unclear. We investigated whether CAR is associated with in-hospital mortality and temporary pacemaker requirement (TPR) in hospitalized AV block. *Materials and Methods*: The study included 698 adults hospitalized with second-degree or higher AV block between January 2012 and July 2020. CAR was calculated from admission C-reactive protein and albumin levels. The primary endpoint was in-hospital mortality, and the secondary endpoint was TPR. *Results*: In-hospital mortality occurred in 14.5% of patients, and TPR occurred in 33.7%. CAR was higher in non-survivors than survivors (0.51 [0.13–1.85] vs. 0.13 [0.05–0.45], *p* < 0.001) and in patients with TPR than those without TPR (0.29 [0.09–1.10] vs. 0.13 [0.05–0.41], *p* < 0.001). Mortality increased across CAR quartiles from 3.8% to 27.7%, and TPR increased from 19.7% to 48.0%. In adjusted models, ln(1 + CAR) was independently associated with mortality (OR 2.14, 95% CI 1.44–3.19; *p* < 0.001) and TPR (OR 1.99, 95% CI 1.38–2.86; *p* < 0.001). Adding CAR modestly improved discrimination for mortality (0.751 to 0.782; *p* = 0.020) and TPR (0.623 to 0.671; *p* < 0.001), without clear superiority over its individual components. *Conclusions*: CAR was independently associated with in-hospital mortality and TPR in hospitalized AV block.

## 1. Introduction

Atrioventricular (AV) block represents a heterogeneous spectrum of conduction system disease, ranging from transient and potentially reversible AV conduction delay to persistent high-grade block requiring urgent pacing support or permanent pacemaker implantation (PPI). Clinical management is primarily guided by electrocardiographic phenotype, symptom burden, hemodynamic status, the presence of reversible causes, and guideline-based indications for temporary or permanent pacing [1,2].

Patients hospitalized with AV block often constitute a high-risk population because advanced age, structural heart disease, renal dysfunction, heart failure (HF), anemia, metabolic disturbances, and drug-related conduction slowing may coexist with the conduction disorder itself. Therefore, short-term outcomes in this setting may not be explained solely by the anatomical level or electrocardiographic type of AV block. Temporary pacemaker implantation may serve not only as a therapeutic intervention but also as a marker of acute clinical instability and urgent need for pacing support [1,2,3].

Systemic inflammation and impaired nutritional status are increasingly recognized as important determinants of adverse outcomes in cardiovascular (CV) disease. C-reactive protein (CRP) is a widely available acute-phase reactant that reflects systemic inflammatory burden and has been associated with CV risk and adverse events [4]. Albumin, in contrast, is a negative acute-phase reactant and a marker of nutritional status, inflammatory activity, frailty, hepatic synthetic function, and systemic disease severity [5]. Because these two biomarkers reflect biologically complementary pathways, their combined assessment may provide more integrated prognostic information than either marker alone.

The C-reactive protein-to-albumin ratio (CAR) is a simple composite index that incorporates both inflammatory activation and nutritional–inflammatory vulnerability. Previous studies have reported that elevated CAR is associated with mortality and adverse clinical outcomes in several CV conditions, including acute and chronic HF, cardiorenal syndrome, acute coronary syndromes, ST-segment elevation myocardial infarction, non-ST-segment elevation myocardial infarction, transcatheter aortic valve implantation, valvular heart disease, and chronic kidney disease (CKD) [6,7,8,9,10,11,12,13,14,15,16]. These findings suggest that the CAR may reflect a broader systemic risk phenotype rather than a disease-specific marker.

Despite this growing body of evidence, the prognostic value of the CAR in patients hospitalized with AV block remains insufficiently defined. This represents an important gap because AV block is commonly approached as an electrical conduction disorder, whereas clinical outcomes may also be influenced by systemic inflammation, nutritional status, comorbidity burden, and acute physiologic vulnerability. Identifying a simple and readily available biomarker that captures this systemic risk may improve early risk stratification in hospitalized patients with AV block.

Therefore, the present study aimed to investigate the association between the CAR and adverse in-hospital outcomes in patients hospitalized with AV block. The primary objective was to determine whether the CAR is independently associated with in-hospital mortality. The secondary objective was to evaluate its association with temporary pacemaker requirement (TPR) as a clinically relevant marker of acute pacing need. We also assessed whether the addition of the CAR provides incremental discriminatory value beyond conventional clinical risk factors and AV block phenotype.

## 2. Methods

### 2.1. Study Design and Population

This single-center, retrospective observational study included patients hospitalized with AV block at a tertiary heart center between January 2012 and July 2020. The study population was identified by screening institutional electronic medical records for patients admitted with second-degree or higher AV block during the index hospitalization.

Patients aged 18 years or older were eligible for inclusion if they were hospitalized with clinically significant AV conduction disease. Eligible electrocardiographic presentations included second-degree AV block, including Mobitz type I, Mobitz type II, and 2:1 AV block; third-degree or complete AV block; and atrial fibrillation (AF) with a slow and regular ventricular response compatible with advanced AV conduction disturbance. In patients with permanent AF, AV block was defined as a ventricular rate below 60 beats/min with a regular ventricular rhythm (equal R–R intervals) on the admission ECG. Patients were excluded if AV block occurred in the setting of acute myocardial infarction, vasovagal reaction, sick sinus syndrome, cardiac surgery, percutaneous cardiac intervention, radiofrequency ablation, or digoxin toxicity. Patients with active infection, active malignancy, chronic inflammatory or autoimmune disease, severe hepatic disease or cirrhosis, or those receiving immunosuppressive therapy or chronic corticosteroid treatment were also excluded. Digoxin toxicity was defined as a serum digoxin level above 2 nmol/L accompanied by clinical symptoms compatible with digoxin toxicity, including nausea, vomiting, or color vision disturbances, or as a serum digoxin level above 2.5 nmol/L regardless of symptoms. Patients with incomplete clinical or outcome data and four patients younger than 18 years were excluded. After applying the eligibility criteria, 698 patients were included in the final analysis.

The study was conducted in accordance with the principles of the Declaration of Helsinki and was approved by the Ethics Committee of Haydarpaşa Numune Training and Research Hospital, University of Health Sciences, Istanbul, Türkiye (approval number: HNEAH-KAEK 2020/KK/223). Because of the retrospective design of the study and the use of anonymized patient data, the requirement for written informed consent was waived by the ethics committee.

### 2.2. Data Collection, Definitions, and Measurements

Demographic characteristics, comorbid diseases, baseline clinical features, admission electrocardiographic findings, laboratory parameters, echocardiographic measurements, medication history, pacemaker procedures, and in-hospital outcomes were obtained retrospectively from institutional electronic medical records. When necessary, national health system records were also reviewed to verify clinical information. Heart rate and systolic blood pressure were recorded at initial hospital presentation. Time from admission to permanent pacemaker implantation was evaluated only in patients who underwent implantation during the index hospitalization.

Coronary artery disease (CAD) was defined as a documented history of CAD, previous myocardial infarction, prior percutaneous coronary intervention, or coronary artery bypass grafting. CKD was identified from a documented pre-existing diagnosis, supported by an admission eGFR < 60 mL/min/1.73 m^2^. Heart failure was identified from a documented pre-existing diagnosis, with reduced systolic function confirmed by an LVEF < 40% on the initial echocardiographic assessment during the index hospitalization. Anemia was defined as a hemoglobin level below 12 g/dL. Ischemic stroke was defined as a documented previous history of ischemic cerebrovascular event.

The electrocardiogram obtained at hospital admission was used to classify the type of AV block. Complete AV block and 2:1 AV block were evaluated separately in the analyses because of their potential association with clinical instability and pacing requirement.

The first blood draw at hospital admission served as the baseline time point for biomarker assessment. Laboratory values were obtained from these samples, which were collected before temporary pacemaker insertion in all patients who underwent temporary pacing. Complete blood count, serum creatinine, urea, electrolytes, CRP, serum albumin, and other available biochemical parameters were recorded. Serum CRP and albumin were measured using the UniCel DxC 800 system (Beckman Coulter, Brea, CA, USA) and the ARCHITECT c8000 system (Abbott Laboratories, Abbott Park, IL, USA), respectively. CAR (mg/g) was calculated by dividing CRP (mg/dL) by albumin (g/dL).

Transthoracic echocardiographic measurements were obtained from the first echocardiographic evaluation performed during the index hospitalization.

### 2.3. Medication Assessment and Pacemaker Procedures

Baseline medication history was recorded from admission files and electronic medical records. Beta-blocker use included propranolol, carvedilol, metoprolol, atenolol, bisoprolol, and nebivolol. Non-dihydropyridine calcium channel blocker use included verapamil and diltiazem. A composite variable defined as “AV nodal/conduction-slowing drug use” was created and included beta-blockers, non-dihydropyridine calcium channel blockers, digoxin, sotalol, amiodarone, donepezil, and topical beta-blockers. Potentially responsible drugs were discontinued during hospitalization when clinically appropriate.

TPR was defined as a clinically assessed need for temporary pacing that resulted in temporary pacemaker insertion during the index hospitalization. The decision was made by the treating cardiology team according to established clinical indications, taking into account the severity of bradyarrhythmia, hemodynamic status, symptoms, and electrocardiographic findings. The term “requirement” therefore denotes clinically determined need resulting in treatment, rather than an independently adjudicated indication based on standardized study criteria.

PPI during the index hospitalization was recorded. PPI was performed in patients with persistent or recurrent clinically significant AV block after evaluation of reversible causes and discontinuation of potentially responsible drugs when applicable.

### 2.4. Study Endpoints

The primary endpoint was in-hospital all-cause mortality during the index hospitalization. Among non-survivors, the primary causes of death were summarized descriptively, and the timing of death was categorized as ≤48 h, >48 h to seven days, or >seven days after admission. The secondary endpoint was TPR during the index hospitalization. PPI was evaluated as an additional procedural characteristic but was not considered a primary outcome of the present study.

### 2.5. Statistical Analysis

Continuous variables were assessed for normality using visual methods and the Kolmogorov–Smirnov test. Normally distributed continuous variables were presented as mean ± standard deviation, whereas non-normally distributed variables were presented as median with interquartile range. Categorical variables were expressed as numbers and percentages.

Patients were primarily compared according to in-hospital mortality status and the temporary pacemaker requirement. Continuous variables were compared using the independent samples Student’s *t*-test or the Mann–Whitney U test, as appropriate. Categorical variables were compared using Pearson’s chi-square test or Fisher’s exact test. An exploratory subgroup analysis was performed among patients who required temporary pacing, comparing admission CAR between survivors and non-survivors using the Mann–Whitney U test.

The CAR was evaluated both as a continuous variable and according to quartiles. Because its distribution was right-skewed, ln(1 + CAR) was used in regression-based analyses. The lowest three quartiles and the highest quartile were also compared in supplementary analyses to evaluate the association of high CAR with clinical outcomes.

Univariable and multivariable logistic regression analyses were performed to evaluate the association between the CAR and study endpoints. The primary endpoint was in-hospital all-cause mortality, and the secondary endpoint was TPR. Regression results were reported as odds ratios with 95% confidence intervals.

For in-hospital mortality, the main clinical model included age, sex, hypertension, diabetes mellitus, CKD, CAD, HF, anemia, and ln(1 + CAR). Model 2 additionally included complete AV block. Temporary pacemaker requirement was not included as a covariate in the revised mortality models because it was a post-admission intervention. Model 3 was a sensitivity analysis including ln(1 + CAR), age, sex, LVEF, eGFR, and hemoglobin. CKD, heart failure, and binary anemia status were omitted from this model to avoid overlap with the corresponding continuous measurements. An extended mortality model (Model 4) additionally adjusted Model 2 for admission heart rate, systolic blood pressure, and lactate. Heart rate and systolic blood pressure were modeled per 10 bpm and 10 mmHg increase, respectively, and lactate per 1 mmol/L increase. All four mortality models included 698 patients with complete data for the respective model variables, including 101 in-hospital deaths. Multicollinearity among the covariates in each mortality model was assessed using variance inflation factors (VIFs) and tolerance values. Internal validation of the four mortality models was performed using 1000 bootstrap resamples, with each model refitted in every resample. Optimism was estimated by comparing model performance in each bootstrap sample with its performance in the original cohort. Optimism-corrected AUCs and calibration slopes were calculated.

For TPR, the main clinical model included age, sex, hypertension, diabetes mellitus, CKD, CAD, HF, anemia, and ln(1 + CAR). A second model additionally included complete AV block and 2:1 AV block.

Because the CAR is mathematically derived from CRP and albumin, these individual components were not entered simultaneously with the ratio in the same multivariable regression models to avoid collinearity. Additional sensitivity analyses were performed after adjustment for AV nodal/conduction-slowing drug use.

Receiver operating characteristic (ROC) curve analyses were performed to evaluate the discriminatory performance of CAR, CRP, and albumin for in-hospital mortality and TPR. AUCs were reported with 95% confidence intervals. Youden-derived cut-off values, sensitivity, and specificity were calculated; however, because these thresholds were derived and evaluated in the same cohort, they were considered exploratory rather than validated clinical decision thresholds.

To compare the incremental discriminatory performance of CAR with its individual components, the same clinical models were supplemented separately with ln(1 + CAR), ln(1 + CRP), albumin, or ln(1 + CRP) and albumin entered simultaneously. Comparisons within each model set were performed using identical complete-case populations. Changes in AUC were reported with 95% confidence intervals.

Model performance was further assessed using calibration intercepts and slopes and Brier scores. Internal validation was performed using 1000 nonparametric bootstrap resamples, with each model refitted in every bootstrap sample. Optimism was estimated by comparing model performance in each bootstrap sample with its performance in the original dataset, and optimism-corrected AUCs, calibration measures, and Brier scores were calculated. Decision-curve analysis was performed to assess net clinical benefit across a range of threshold probabilities.

A *p* value < 0.05 was considered statistically significant. Statistical analyses were performed using IBM SPSS Statistics for Windows, Version 31.0 (IBM Corp., Armonk, NY, USA).

## 3. Results

A total of 698 patients hospitalized with AV block were included. The median age of the study population was 77.0 years [67.0–84.8], and 312 patients (44.7%) were male. During the index hospitalization, 101 patients (14.5%) died, whereas 597 patients (85.5%) survived. The most frequent primary causes of death were cardiogenic shock or progressive heart failure (31 patients, 30.7%), hospital-acquired infection or sepsis (22 patients, 21.8%), and arrhythmic death (14 patients, 13.9%). Of the 101 deaths, 24 (23.8%) occurred within 48 h of admission, 38 (37.6%) occurred after 48 h and within seven days, and 39 (38.6%) occurred after seven days (Supplementary Table S1). Among the 101 patients who died, 45 had undergone temporary pacing and 56 had undergone permanent pacemaker implantation before death; these groups were not mutually exclusive.

Baseline clinical, laboratory, medication, electrocardiographic, and procedural characteristics according to in-hospital mortality are presented in Table 1. Non-survivors were older than survivors and had a higher prevalence of CKD, CAD, HF, and anemia. Diabetes mellitus was less frequent among non-survivors. LVEF was lower in non-survivors, whereas white blood cell count, creatinine, CRP, and the CAR were significantly higher. Albumin levels were significantly lower among non-survivors. Non-survivors also had lower admission heart rates (38 [32–45] vs. 43 [37–52] bpm), lower systolic blood pressures (122 [98–146] vs. 138 [118–158] mmHg), and higher lactate levels (2.60 [1.60–4.30] vs. 1.40 [1.00–2.00] mmol/L) than survivors (all *p* < 0.001) (Table 1). Regarding medication history, any beta-blocker use was less frequent among non-survivors, whereas the overall rate of AV nodal/conduction-slowing drug use did not differ significantly between survivors and non-survivors. Complete AV block and TPR were more frequent among non-survivors, whereas 2:1 AV block and PPI were less frequent in this group.

Temporary pacemaker support was required in 235 patients (33.7%), whereas 463 patients (66.3%) did not require temporary pacing during the index hospitalization. Baseline characteristics according to TPR are shown in Table 2. Patients requiring temporary pacemaker support were older and had a higher prevalence of hypertension and CKD. These patients also had lower admission heart rates (36 [30–42] vs. 45 [38–54] bpm), lower systolic blood pressures (124 [100–146] vs. 142 [124–162] mmHg), and higher lactate levels (2.60 [1.40–3.20] vs. 1.30 [1.00–1.80] mmol/L) than those not requiring temporary pacing (all *p* < 0.001) (Table 2). Hemoglobin and eGFR rate were lower in the temporary pacemaker group, whereas white blood cell count, creatinine, potassium, aspartate aminotransferase (AST), alanine aminotransferase (ALT), CRP, and the CAR were significantly higher. Albumin levels were significantly lower among patients who required temporary pacing, whereas thyroid-stimulating hormone levels did not differ significantly between groups. Regarding medication history, any beta-blocker use and overall AV nodal/conduction-slowing drug use were more frequent in patients requiring temporary pacemaker support. Complete AV block was substantially more common in the temporary pacemaker group, whereas 2:1 AV block was less frequent. PPI did not differ significantly according to TPR. Among patients who underwent PPI during the index hospitalization, the median time from admission to implantation was 4 [2–6] days. This interval was shorter in patients requiring temporary pacing than in those not requiring temporary pacing (3 [2–5] vs. 4 [2–7] days; *p* = 0.004) and did not differ significantly between survivors and non-survivors (4 [2–6] vs. 3 [1–6] days; *p* = 0.152) (Table 1). Within the temporary pacing group, admission CAR was higher in the 45 non-survivors than in the 190 survivors (median [IQR]: 0.947 [0.191–1.887] vs. 0.255 [0.067–0.893]; *p* < 0.001).

Patients were stratified into quartiles according to CAR values. A progressive increase in adverse in-hospital outcomes was observed across increasing quartiles of the CAR. In-hospital mortality increased from 3.8% in the lowest quartile to 27.7% in the highest quartile. Similarly, TPR increased from 19.7% in the lowest quartile to 48.0% in the highest quartile. These findings demonstrated a stepwise relationship between higher CAR and both in-hospital mortality and TPR (both *p* < 0.001; Table 3).

Multivariable logistic regression analyses were performed to evaluate the independent association between the CAR and in-hospital mortality. In the main clinical model adjusted for age, sex, hypertension, diabetes mellitus, CKD, CAD, HF, and anemia, ln(1 + CAR) was independently associated with in-hospital mortality (OR 2.29, 95% CI 1.55–3.40; *p* < 0.001).

After additional adjustment for complete AV block, ln(1 + CAR) remained associated with in-hospital mortality in Model 2 (OR 2.14, 95% CI 1.44–3.19; *p* < 0.001). In Model 3, which included age, sex, LVEF, eGFR, and hemoglobin alongside ln(1 + CAR), the association also remained statistically significant (OR 2.90, 95% CI 1.53–5.51; *p* = 0.001). In Model 4, which additionally adjusted Model 2 for admission heart rate, systolic blood pressure, and lactate, the association was attenuated but remained statistically significant (OR 1.65, 95% CI 1.10–2.47; *p* = 0.015; Table 4).

There was no evidence of substantial multicollinearity in the revised mortality models. VIF values ranged from 1.069 to 1.195 in Model 1, 1.036 to 1.195 in Model 2, 1.040 to 1.327 in Model 3, and 1.094 to 1.226 in Model 4. The minimum tolerance across all four models was 0.754. Bootstrap internal validation yielded optimism-corrected AUCs of 0.757, 0.761, 0.599, and 0.821 for Models 1–4, respectively. The corresponding optimism-corrected calibration slopes were 0.885, 0.878, 0.670, and 0.875. All 1000 bootstrap replicates were successful for each model (Supplementary Table S2).

Multivariable logistic regression analyses were performed to identify independent factors associated with TPR. In the main clinical model adjusted for age, sex, hypertension, diabetes mellitus, CKD, CAD, HF, anemia, and ln(1 + CAR), ln(1 + CAR) was independently associated with TPR (OR 2.25, 95% CI 1.57–3.21; *p* < 0.001). After additional adjustment for AV block phenotype, including complete AV block and 2:1 AV block, the association between ln(1 + CAR) and TPR remained statistically significant (OR 1.99, 95% CI 1.38–2.86; *p* < 0.001). In the same model, complete AV block was also independently associated with TPR (OR 2.61, 95% CI 1.45–4.72; *p* = 0.001), whereas 2:1 AV block was not independently associated with this outcome (Table 5).

ROC analyses were performed to evaluate the discriminatory performance of CAR, CRP, and albumin for in-hospital mortality and TPR. For in-hospital mortality, the stand-alone AUCs were 0.699 for CAR, 0.684 for CRP, and 0.690 for albumin. For TPR, the corresponding AUCs were 0.630, 0.615, and 0.653, respectively. Additional analyses evaluated the incremental discriminatory value of each biomarker using identical complete-case populations within each model set. For in-hospital mortality, the clinical model had an AUC of 0.751 (95% CI 0.701–0.798). Addition of CAR increased the AUC to 0.782 (95% CI 0.738–0.828; ΔAUC 0.031, 95% CI 0.005–0.058; *p* = 0.020), CRP to 0.783 (95% CI 0.735–0.826; ΔAUC 0.032, 95% CI 0.004–0.061; *p* = 0.023), and albumin to 0.774 (95% CI 0.727–0.820; ΔAUC 0.024, 95% CI −0.007–0.055; *p* = 0.142). When CRP and albumin were entered simultaneously, the AUC increased to 0.792 (95% CI 0.746–0.834; ΔAUC 0.041, 95% CI 0.008–0.075; *p* = 0.014).

For TPR, the clinical model had an AUC of 0.623 (95% CI 0.579–0.666). The AUC increased to 0.671 with CAR (95% CI 0.629–0.709; ΔAUC 0.048, 95% CI 0.019–0.074; *p* < 0.001), 0.673 with CRP (95% CI 0.631–0.713; ΔAUC 0.050, 95% CI 0.017–0.081; *p* = 0.002), 0.699 with albumin (95% CI 0.656–0.739; ΔAUC 0.075, 95% CI 0.039–0.112; *p* < 0.001), and 0.711 when CRP and albumin were entered simultaneously (95% CI 0.670–0.751; ΔAUC 0.088, 95% CI 0.049–0.126; *p* < 0.001). In the model additionally incorporating AV block phenotype, the base AUC was 0.712 (95% CI 0.669–0.747). The corresponding AUCs were 0.730 with CAR (ΔAUC 0.018, 95% CI 0.001–0.035; *p* = 0.038), 0.730 with CRP (ΔAUC 0.018, 95% CI −0.000–0.037; *p* = 0.055), 0.749 with albumin (ΔAUC 0.037, 95% CI 0.013–0.060; *p* = 0.004), and 0.756 with CRP plus albumin (ΔAUC 0.044, 95% CI 0.019–0.069; *p* < 0.001) (Table 6).

Bootstrap internal validation showed that optimism-corrected AUCs for the base, CAR, CRP, albumin, and CRP-plus-albumin models were 0.725, 0.758, 0.759, 0.751, and 0.767 for in-hospital mortality; 0.596, 0.647, 0.649, 0.679, and 0.690 for the clinical TPR models; and 0.692, 0.709, 0.709, 0.731, and 0.736 for the TPR models incorporating AV block phenotype, respectively. Optimism-corrected calibration slopes ranged from 0.884 to 0.890 for mortality, from 0.798 to 0.882 for the clinical TPR models, and from 0.881 to 0.899 for the AV-block-phenotype-adjusted TPR models. Corresponding optimism-corrected Brier scores ranged from 0.113 to 0.116, 0.206 to 0.220, and 0.193 to 0.203, respectively. All 1000 bootstrap replicates were successfully completed. Decision-curve analysis showed threshold-dependent differences in net benefit, without consistent superiority of CAR over CRP, albumin, or their combination (Supplementary Table S5 and Supplementary Figure S1).

Additional sensitivity analyses were performed to assess the robustness of the association between the CAR and study endpoints. After additional adjustment for AV nodal/conduction-slowing drug use, ln(1 + CAR) remained associated with in-hospital mortality in the clinical model (OR 1.80, 95% CI 1.28–2.54; *p* < 0.001) and the model additionally adjusted for complete AV block (OR 2.11, 95% CI 1.42–3.15; *p* < 0.001). AV nodal/conduction-slowing drug use itself was not independently associated with in-hospital mortality in these models. Similarly, the association between ln(1 + CAR) and TPR persisted after adjustment for AV nodal/conduction-slowing drug use. In the clinical model, ln(1 + CAR) remained independently associated with TPR (OR 1.81, 95% CI 1.29–2.53; *p* < 0.001). This association also remained significant after additional adjustment for complete AV block and 2:1 AV block (OR 1.61, 95% CI 1.16–2.23; *p* = 0.005). In this model, complete AV block remained independently associated with TPR, whereas AV nodal/conduction-slowing drug use was not independently associated with temporary pacing. In supplementary analyses using the highest quartile of the CAR as a categorical variable, high CAR was independently associated with both in-hospital mortality (OR 2.54, 95% CI 1.59–4.08; *p* < 0.001) and TPR (OR 2.50, 95% CI 1.71–3.65; *p* < 0.001) (Supplementary Tables S3 and S4). These findings were consistent with the main regression analyses using ln(1 + CAR). Overall, the association between the CAR and adverse in-hospital outcomes remained consistent across clinical models, AV block phenotype-adjusted models, and medication-adjusted sensitivity analyses.

## 4. Discussion

In this single-center cohort of patients hospitalized with AV block, the principal findings were as follows. First, a higher CAR was independently associated with in-hospital mortality. Second, CAR was also independently associated with TPR, a clinically relevant marker of acute pacing need and in-hospital instability. Third, these associations persisted after adjustment for major demographic and clinical variables, AV block phenotype, and AV nodal/conduction-slowing drug use. Fourth, although the stand-alone discriminatory performance of CAR was moderate, its addition to clinical prediction models improved discrimination for both in-hospital mortality and TPR. However, additional comparative analyses showed that CAR did not provide clear discriminatory superiority over CRP or albumin alone, with albumin providing greater incremental discrimination for TPR.

AV block is primarily approached as an electrical conduction disorder, and clinical decision-making is generally guided by electrocardiographic phenotype, symptom burden, hemodynamic status, reversibility, and guideline-based pacing indications [1,2]. However, the present findings suggest that short-term outcomes in hospitalized patients with AV block are not determined solely by the type of conduction abnormality. In our cohort, non-survivors were older and had a higher burden of CKD, CAD, HF, anemia, lower LVEF, higher inflammatory burden, and lower albumin levels. These findings indicate that in-hospital mortality in AV block reflects not only conduction system disease but also systemic vulnerability, comorbidity burden, inflammatory activation, and impaired physiologic reserve.

The relatively high in-hospital mortality rate observed in this cohort should be interpreted in the context of a high-risk hospitalized AV block population rather than a stable elective pacemaker population. Patients requiring temporary pacing represent a clinically vulnerable group, and previous data have shown that temporary transvenous pacing is associated with substantial in-hospital morbidity and mortality [3,17,18]. In our study, TPR was more frequent among non-survivors, and complete AV block was also more common in this group. After additional adjustment for admission heart rate, systolic blood pressure, and lactate, the association between CAR and mortality was attenuated but remained statistically significant. This attenuation suggests that the association may partly reflect illness severity at presentation. The remaining association should not be interpreted as evidence of AV-block-specific risk, as CAR may also capture broader inflammatory, nutritional, and systemic vulnerability. The distribution of causes and timing of death further supports this interpretation. Cardiogenic shock or progressive heart failure, hospital-acquired infection or sepsis, and arrhythmic death were the leading causes, while 38.6% of deaths occurred more than seven days after admission. These findings suggest that the observed mortality reflects both cardiovascular disease and complications developing during hospitalization, rather than the conduction disturbance alone.

Bootstrap validation showed that Model 4 retained discrimination after optimism correction, with a corrected AUC of 0.821, although its corrected calibration slope of 0.875 indicated some overfitting. Model 3 showed greater optimism and limited corrected discrimination (AUC 0.599; calibration slope 0.670), warranting cautious interpretation of this sensitivity analysis. These performance estimates apply to the complete multivariable models rather than CAR alone, and external validation remains necessary before clinical predictive use.

The biological plausibility of CAR as a prognostic marker is supported by the complementary roles of its two components. CRP is a positive acute-phase reactant reflecting systemic inflammatory activation, cytokine signaling, tissue injury, ischemic stress, and acute illness severity [4]. Albumin is a negative acute-phase reactant and is influenced by nutritional status, systemic inflammation, frailty, hepatic synthetic function, vascular permeability, oxidative stress, and chronic disease burden [5]. The combined use of CRP and albumin as a ratio was originally proposed as an integrated marker of acute illness severity and prognosis, and subsequent studies in critically ill populations supported its prognostic value [19,20]. Therefore, a higher CAR may identify patients in whom inflammatory activation is accompanied by reduced nutritional–inflammatory reserve.

Previous studies have reported the prognostic value of CAR across several CV populations. Elevated CAR has been associated with adverse outcomes in acute HF, chronic HF with reduced ejection fraction, cardiorenal syndrome, CKD, acute coronary syndromes, ST-segment elevation myocardial infarction, non-ST-segment elevation myocardial infarction, transcatheter aortic valve implantation, and valvular heart disease [6,7,8,9,10,11,12,13,14,15,16]. Population-based data have also linked CAR with cardiovascular outcomes and mortality [21]. Additional studies have linked CAR with CAD severity, thrombotic or reperfusion-related complications after myocardial infarction, and adverse outcomes after coronary intervention [22,23,24]. The present study extends this literature to patients hospitalized with AV block, a population in which the clinical role of inflammatory–nutritional biomarkers remains insufficiently defined.

One of the most clinically relevant findings of the present study was the stepwise increase in in-hospital mortality across CAR quartiles. Mortality increased from 3.8% in the lowest quartile to 27.7% in the highest quartile. This gradient supports a graded association between inflammatory–nutritional burden and adverse in-hospital outcome. This association remained significant after adjustment for age, sex, hypertension, diabetes mellitus, CKD, CAD, HF, anemia, and complete AV block. These findings support an association between CAR and mortality after adjustment for the measured demographic, clinical, and AV-block characteristics, although residual confounding remains possible.

Several mechanisms may explain the association between higher CAR and in-hospital mortality in patients with AV block. Systemic inflammation may reflect acute illness severity, ischemic burden, renal dysfunction, decompensated HF, or overall systemic vulnerability, each of which can impair hemodynamic tolerance to bradyarrhythmia. Hypoalbuminemia may represent impaired nutritional reserve, frailty, vascular dysfunction, and limited physiologic reserve. In addition, inflammation, oxidative stress, endothelial dysfunction, renal impairment, electrolyte disturbances, and fibrotic remodeling may influence myocardial tissue and the His-Purkinje system. Previous studies have linked reduced kidney function and metabolic disturbances with cardiac conduction defects, and hyperkalemia has long been recognized as a potential contributor to atrioventricular and intraventricular conduction abnormalities [25,26]. Experimental and translational data also support the role of myocardial fibrosis and transforming growth factor-β-related pathways in progressive conduction disease [27,28]. Although our study cannot establish causality, these mechanisms provide a biologically plausible framework linking elevated CAR with adverse outcomes in AV block.

Another important finding was the independent association between CAR and TPR. Temporary pacing in AV block is usually determined by hemodynamic instability, severity of bradycardia, escape rhythm characteristics, symptoms, electrocardiographic findings, and the likelihood of reversibility [1,2]. In our cohort, patients with TPR had a broader adverse laboratory and clinical profile, including higher WBC count, creatinine, potassium, AST, ALT, CRP, and CAR, together with lower Hb, eGFR, and albumin levels, as well as a higher prevalence of complete AV block. TPR also increased progressively across CAR quartiles, from 19.7% in the lowest quartile to 48.0% in the highest quartile. These findings suggest that TPR was accompanied by systemic inflammatory, renal-metabolic, and overall illness-severity signals rather than by electrocardiographic severity alone. Higher potassium levels and worse renal function may be particularly relevant in AV block, because renal-metabolic and electrolyte disturbances can aggravate conduction abnormalities and reduce hemodynamic tolerance to bradyarrhythmia. However, these laboratory differences should be interpreted as descriptive markers of clinical vulnerability rather than independent determinants of TPR.

The association between CAR and TPR remained significant after adjustment for complete AV block and 2:1 AV block. Complete AV block was independently associated with TPR, as expected. However, the persistence of the CAR association after adjustment for AV block phenotype suggests that inflammatory–nutritional burden may provide additional information beyond the surface electrocardiogram. In clinical practice, two patients with similar AV block phenotype may differ substantially in hemodynamic reserve, inflammatory state, renal function, and systemic illness burden; CAR may help capture this difference.

Medication-related AV block is an important consideration in this population. Previous studies have shown that AV block may regress after discontinuation of AV nodal blocking drugs; however, persistence or recurrence of clinically significant AV block is not uncommon, suggesting that drug exposure may unmask underlying conduction system disease rather than represent the sole mechanism in all patients [29,30,31,32]. In the present study, beta-blocker use and overall AV nodal/conduction-slowing drug use were more frequent among patients requiring temporary pacemaker support. Nevertheless, CAR remained independently associated with both in-hospital mortality and TPR after additional adjustment for AV nodal/conduction-slowing drug use. This finding supports the interpretation that the prognostic value of CAR was not merely explained by baseline exposure to conduction-slowing medications.

The lower rate of PPI among non-survivors deserves careful interpretation. This finding should not be interpreted as evidence that PPI is protective or harmful. Rather, it likely reflects survivorship and treatment eligibility bias. Patients who died during hospitalization may not have survived long enough, or may not have been clinically stable enough, to undergo PPI. Therefore, PPI should be interpreted as a procedural characteristic influenced by clinical course, reversibility assessment, and survival to intervention rather than as a direct determinant of mortality in this retrospective dataset.

The ROC analyses provide further context regarding the discriminatory performance of CAR and its individual components. The stand-alone discriminatory performance of CAR was modest and was numerically similar to that of CRP and albumin. In the clinical prediction models, addition of CAR and CRP resulted in similar improvements in discrimination for in-hospital mortality, whereas the combination of CRP and albumin produced the largest numerical increase in AUC. For TPR, albumin and the combined CRP-plus-albumin model provided greater incremental discrimination than CAR, both in the clinical model and after incorporation of AV block phenotype. Internal bootstrap validation showed that these patterns were generally maintained after optimism correction. Decision-curve analysis demonstrated threshold-dependent differences in net benefit, without consistent clinical-utility superiority of CAR over CRP, albumin, or their combination. Taken together, these findings indicate that CAR does not demonstrate clear predictive superiority over its individual components or their combined inclusion in a multivariable model. Its potential value should therefore be interpreted as that of a simple composite marker of inflammatory–nutritional vulnerability rather than as a replacement for CRP, albumin, or established clinical assessment.

The Youden-derived cut-off values should also be interpreted cautiously. Because these thresholds were derived and evaluated within the same cohort, they are exploratory and may overestimate performance. They should not be considered validated clinical decision thresholds, and external validation is required before their use in clinical risk stratification or pacing-related decision-making.

From a practical standpoint, CAR has several advantages. It is inexpensive, rapidly available, easily calculated, and derived from routinely measured laboratory parameters. In hospitalized patients with AV block, CAR may help clinicians identify individuals with higher inflammatory–nutritional vulnerability who may require closer monitoring, careful assessment of reversible causes, and earlier planning of pacing strategy. However, CAR should complement, not replace, clinical judgment, hemodynamic assessment, electrocardiographic evaluation, and guideline-based pacing indications [1,2].

### Study Limitations

The present study has several limitations. First, this was a single-center retrospective study; therefore, the findings require confirmation in prospective multicenter cohorts. Second, CAR was calculated from laboratory values obtained at admission, and serial changes in CRP, albumin, and CAR were not evaluated. Third, TPR reflected a clinical management decision influenced by hemodynamic status, symptoms, rhythm characteristics, physician judgment, and institutional practice rather than an independently adjudicated endpoint. Although admission blood sampling preceded temporary pacemaker insertion in all patients who underwent temporary pacing, exact sampling-to-implantation intervals were unavailable. The observed associations therefore should not be interpreted as evidence that CAR prospectively predicts the need for temporary pacing or should influence pacing decisions. Although the extended mortality model incorporated admission heart rate, systolic blood pressure, and lactate, other indicators of acute clinical severity, including syncope, escape-rhythm characteristics, vasoactive therapy, ventilatory support, and reversible etiologies, were not consistently available. Residual confounding therefore remains possible. In addition, the biomarker models were internally validated using bootstrap resampling but were not externally validated. The Youden-derived cut-off values were derived and evaluated in the same cohort and should therefore be considered exploratory. Decision-curve analysis did not demonstrate consistent superiority of CAR over CRP, albumin, or their combination across threshold probabilities. Accordingly, the present study does not establish clinical utility or superiority of CAR over its individual components. Finally, because of the observational design, causal inference cannot be made. Despite these limitations, this study included a relatively large cohort of patients hospitalized with AV block and demonstrated consistent associations between admission CAR and both in-hospital mortality and TPR across several adjusted and sensitivity analyses. These findings support further evaluation of CAR as a simple marker of inflammatory–nutritional vulnerability, while prospective external validation is required before any predictive or clinical application.

## 5. Conclusions

Higher admission CAR was independently associated with in-hospital mortality and TPR in patients hospitalized with AV block. However, CAR did not demonstrate clear discriminatory or clinical-utility superiority over CRP, albumin, or their combined use in prediction models. CAR should therefore be interpreted as a simple composite marker of inflammatory–nutritional vulnerability rather than as an established clinical decision tool. Prospective external validation is required before its predictive or clinical utility can be determined.

## Figures and Tables

**Table 1:** Baseline clinical, laboratory, medication, electrocardiographic, and procedural characteristics of study population.

Variable	Overall (*n* = 698)	Survivors (*n* = 597)	Non-Survivors (*n* = 101)	*p* Value
**Demographic and clinical characteristics**				
Age, years	77.00 [67.00–84.75]	77.00 [66.00-84.00]	81.00 [71.00–89.00]	**0.003**
Male sex	312 (44.7)	260 (43.6)	52 (51.5)	0.142
Hypertension	407 (58.3)	354 (59.3)	53 (52.5)	0.237
Diabetes mellitus	190 (27.2)	171 (28.6)	19 (18.8)	**0.044**
Hyperlipidemia	83 (11.9)	72 (12.1)	11 (10.9)	0.745
Chronic kidney disease	126 (18.1)	90 (15.1)	36 (35.6)	**<0.001**
Coronary artery disease	218 (31.2)	178 (29.8)	40 (39.6)	**0.044**
Previous CABG	81 (11.6)	71 (11.9)	10 (9.9)	0.577
Heart failure	112 (16.0)	76 (12.7)	36 (35.6)	**<0.001**
Ischemic stroke	27 (3.9)	22 (3.7)	5 (5.0)	0.531
COPD	24 (3.4)	21 (3.5)	3 (3.0)	1.000
Anemia	256 (36.7)	200 (33.5)	56 (55.4)	**<0.001**
**Medication history**				
ACE inhibitor	113 (16.2)	98 (16.4)	15 (14.9)	0.693
Angiotensin receptor blocker	52 (7.4)	47 (7.9)	5 (5.0)	0.301
Mad honey exposure	14 (2.0)	14 (2.3)	0 (0.0)	0.240
Any beta-blocker use	218 (31.2)	196 (32.8)	22 (21.8)	**0.027**
Non-dihydropyridine calcium channel blocker	22 (3.2)	19 (3.2)	3 (3.0)	1.000
Digoxin	25 (3.6)	18 (3.0)	7 (6.9)	0.074
Sotalol	1 (0.1)	0 (0.0)	1 (1.0)	0.145
Amiodarone	11 (1.6)	11 (1.8)	0 (0.0)	0.381
Donepezil	6 (0.9)	5 (0.8)	1 (1.0)	1.000
Any AV nodal/conduction-slowing drug	239 (34.2)	209 (35.0)	30 (29.7)	0.299
**Laboratory and echocardiographic parameters**				
LVEF, %	60.00 [50.00–60.00]	60.00 [50.00–60.00]	55.00 [40.00–60.00]	**0.004**
Hemoglobin, g/dL	12.10 [11.00–13.40]	12.30 [11.00–13.40]	11.80 [10.45–13.35]	0.102
WBC count, ×10^3^/µL	8.17 [6.59–10.30]	8.05 [6.50–10.00]	8.98 [7.50–11.61]	**0.001**
Creatinine, mg/dL	0.90 [0.78–1.20]	0.90 [0.77–1.15]	1.10 [0.86–1.58]	**<0.001**
eGFR, mL/min/1.73 m^2^	68.54 [46.00–86.00]	69.85 [47.00–86.23]	53.22 [39.20–73.41]	**0.030**
Potassium, mmol/L	4.50 [4.10–5.10]	4.50 [4.10–5.20]	4.40 [4.10–4.80]	0.123
TSH, mIU/L	1.37 [0.80–2.60]	1.34 [0.80–2.60]	1.48 [0.85–2.59]	0.830
AST, U/L	21.00 [16.00–34.00]	21.00 [16.00–33.00]	23.00 [16.00–39.00]	0.183
ALT, U/L	20.00 [14.00–33.00]	20.00 [14.00–32.00]	22.00 [12.00–41.00]	0.765
Albumin, g/dL	3.70 [3.40–4.06]	3.80 [3.46–4.10]	3.45 [3.10–3.70]	**<0.001**
CRP, mg/dL	0.60 [0.20–2.30]	0.50 [0.20–1.62]	2.10 [0.50–6.47]	**<0.001**
C-reactive protein-to-albumin ratio, mg/g	0.15 [0.06–0.62]	0.13 [0.05–0.45]	0.51 [0.13–1.85]	**<0.001**
Admission heart rate, bpm	42 [36–51]	43 [37–52]	38 [32–45]	**<0.001**
Admission systolic blood pressure, mmHg	136 [115–157]	138 [118–158]	122 [98–146]	**<0.001**
Admission lactate, mmol/L	1.50 [1.10–2.20]	1.40 [1.00–2.00]	2.60 [1.60–4.30]	**<0.001**
**Electrocardiographic and procedural characteristics**				
Complete atrioventricular block	440 (63.0)	361 (60.5)	79 (78.2)	**<0.001**
2:1 atrioventricular block	216 (31.0)	197 (33.0)	19 (18.8)	**0.005**
Mobitz Type I atrioventricular block	12 (1.7)	12 (2.0)	0 (0)	0.232
Mobitz Type II atrioventricular block	30 (4.3)	27 (4.5)	3 (3)	0.604
Temporary pacemaker	235 (33.7)	190 (31.8)	45 (44.6)	**0.011**
Permanent pacemaker	492 (70.5)	436 (73.0)	56 (55.4)	**<0.001**
Time from admission to PPI, days *	4 [2–6]	4 [2–6]	3 [1–6]	0.152

Data are presented as median [interquartile range] or *n* (%). ACE, angiotensin-converting enzyme; ALT, alanine aminotransferase; AST, aspartate aminotransferase; AV, atrioventricular; CABG, coronary artery bypass grafting; COPD, chronic obstructive pulmonary disease; CRP, C-reactive protein; eGFR, estimated glomerular filtration rate; LVEF, left ventricular ejection fraction; TSH, thyroid-stimulating hormone; WBC, white blood cell count. * Evaluated only in patients who underwent permanent pacemaker implantation during the index hospitalization.

**Table 2:** Baseline clinical, laboratory, medication, electrocardiographic, and procedural characteristics according to temporary pacemaker requirement.

Variable	Overall (*n* = 698)	No Temporary PM (*n* = 463)	Temporary PM (*n* = 235)	*p* Value
**Demographic and clinical characteristics**				
Age, years	77.00 [67.00–84.75]	76.00 [66.00–84.00]	78.00 [71.50–85.00]	**0.020**
Male sex	312 (44.7)	215 (46.4)	97 (41.3)	0.198
Hypertension	407 (58.3)	249 (53.8)	158 (67.2)	**<0.001**
Diabetes mellitus	190 (27.2)	118 (25.5)	72 (30.6)	0.149
Hyperlipidemia	83 (11.9)	56 (12.1)	27 (11.5)	0.849
Chronic kidney disease	126 (18.1)	73 (15.8)	53 (22.6)	**0.019**
Coronary artery disease	218 (31.2)	135 (29.2)	83 (35.3)	0.072
Previous CABG	81 (11.6)	59 (12.7)	22 (9.4)	0.181
Heart failure	112 (16.0)	68 (14.7)	44 (18.7)	0.146
Ischemic stroke	27 (3.9)	14 (3.0)	13 (5.5)	0.106
COPD	24 (3.4)	17 (3.7)	7 (3.0)	0.717
Anemia	256 (36.7)	166 (35.9)	90 (38.3)	0.530
Admission heart rate, bpm	42 [36–51]	45 [38–54]	36 [30–42]	**<0.001**
Admission systolic blood pressure, mmHg	136 [115–157]	142 [124–162]	124 [100–146]	**<0.001**
Admission lactate, mmol/L	1.50 [1.10–2.20]	1.30 [1.00–1.80]	2.60 [1.40–3.20]	**<0.001**
**Medication history**				
ACE inhibitor	113 (16.2)	67 (14.5)	46 (19.6)	**0.090**
Angiotensin receptor blocker	52 (7.4)	29 (6.3)	23 (9.8)	**0.099**
Mad honey exposure	14 (2.0)	10 (2.2)	4 (1.7)	0.782
Any beta-blocker use	218 (31.2)	131 (28.3)	87 (37.0)	**0.018**
Non-dihydropyridine calcium channel blocker	22 (3.2)	16 (3.5)	6 (2.6)	0.510
Digoxin	25 (3.6)	14 (3.0)	11 (4.7)	0.408
Sotalol	1 (0.1)	1 (0.2)	0 (0.0)	1.000
Amiodarone	11 (1.6)	8 (1.7)	3 (1.3)	0.758
Donepezil	6 (0.9)	3 (0.6)	3 (1.3)	0.412
Any AV nodal/conduction-slowing drug	239 (34.2)	145 (31.3)	94 (40)	**0.030**
**Laboratory and echocardiographic parameters**				
LVEF, %	60.00 [50.00–60.00]	60.00 [50.00–60.00]	60.00 [45.00–60.00]	0.347
Hemoglobin, g/dL	12.10 [11.00–13.40]	12.30 [11.20–13.40]	11.80 [10.35–13.10]	**0.001**
WBC count, ×10^3^/µL	8.17 [6.59–10.30]	7.80 [6.44–9.74]	9.01 [7.45–11.27]	**<0.001**
Creatinine, mg/dL	0.90 [0.78–1.20]	0.90 [0.77–1.15]	0.99 [0.80–1.33]	**0.006**
eGFR, mL/min/1.73 m^2^	68.54 [46.00–86.00]	74.00 [49.02–86.70]	61.17 [45.00–81.90]	**0.004**
Potassium, mmol/L	4.50 [4.10–5.10]	4.40 [4.10–4.98]	4.60 [4.10–5.85]	**0.004**
TSH, mIU/L	1.37 [0.80–2.60]	1.40 [0.82–2.59]	1.25 [0.65–2.65]	0.493
AST, U/L	21.00 [16.00–34.00]	20.00 [16.00–28.00]	25.00 [18.00–43.00]	**<0.001**
ALT, U/L	20.00 [14.00–33.00]	19.00 [14.00–28.00]	23.50 [15.00–37.00]	**<0.001**
Albumin, g/dL	3.70 [3.40–4.06]	3.80 [3.50–4.10]	3.50 [3.20–3.90]	**<0.001**
CRP, mg/dL	0.60 [0.20–2.30]	0.50 [0.20–1.45]	1.00 [0.30–3.80]	**<0.001**
C-reactive protein-to-albumin ratio	0.15 [0.06–0.62]	0.13 [0.05–0.41]	0.29 [0.09–1.10]	**<0.001**
**Electrocardiographic and procedural characteristics**				
Complete atrioventricular block	440 (63.0)	245 (52.9)	195 (83.0)	<0.001
2:1 atrioventricular block	216 (31.0)	183 (39.5)	33 (14.0)	<0.001
Mobitz Type I atrioventricular block	12 (1.7)	12 (2.6)	0 (0)	**0.006**
Mobitz Type II atrioventricular block	30 (4.3)	23 (5)	7 (3)	0.215
Permanent pacemaker	492 (70.5)	332 (71.7)	160 (68.1)	0.185
Time from admission to PPI, days *	4 [2–6]	4 [2–7]	3 [2–5]	**0.004**

Data are presented as median [interquartile range] or *n* (%). ACE, angiotensin-converting enzyme; ALT, alanine aminotransferase; AST, aspartate aminotransferase; AV, atrioventricular; CABG, coronary artery bypass grafting; COPD, chronic obstructive pulmonary disease; CRP, C-reactive protein; eGFR, estimated glomerular filtration rate; LVEF, left ventricular ejection fraction; PM, pacemaker; TSH, thyroid-stimulating hormone; WBC, white blood cell count. * Evaluated only in patients who underwent permanent pacemaker implantation during the index hospitalization.

**Table 3:** In-hospital mortality and temporary pacemaker requirement across C-reactive protein-to-albumin ratio quartiles.

C-Reactive Protein-to-Albumin Ratio Quartile	*n*	In-Hospital Death, *n*/*N* (%)	Temporary Pacemaker, *n*/*N* (%)
Q1	183	7/183 (3.8)	36/183 (19.7)
Q2	167	21/167 (12.6)	55/167 (32.9)
Q3	175	25/175 (14.3)	61/175 (34.9)
Q4	173	48/173 (27.7)	83/173 (48.0)
*p* value		**<0.001**	**<0.001**

Data are presented as *n*/*N* (%). Quartiles were defined according to the sample distribution of the C-reactive protein-to-albumin ratio. The cut-off values for quartile classification were 0.056, 0.153, and 0.625.

**Table 4:** Multivariable logistic regression models for in-hospital mortality.

Variable	Model 1 OR (95% CI)	*p* Value	Model 2 OR (95% CI)	*p* Value	Model 3 OR (95% CI)	*p* Value	Model 4 OR (95% CI)	*p* Value
ln(1 + CAR)	2.29 (1.55–3.40)	**<0.001**	2.14 (1.44–3.19)	**<0.001**	2.90 (1.53–5.51)	**0.001**	1.65 (1.10–2.47)	**0.015**
Age, per year	1.02 (1.00–1.04)	0.075	1.02 (1.00–1.04)	0.060	1.00 (0.96–1.04)	0.989	1.01 (0.99–1.03)	0.325
Male sex	1.26 (0.78–2.04)	0.355	1.29 (0.79–2.09)	0.305	1.52 (0.56–4.12)	0.410	1.31 (0.82–2.09)	0.258
Hypertension	0.83 (0.50–1.36)	0.455	0.79 (0.48–1.31)	0.357	—	—	0.81 (0.49–1.35)	0.415
Diabetes mellitus	0.51 (0.28–0.95)	**0.034**	0.52 (0.28–0.96)	**0.037**	—	—	0.52 (0.28–0.97)	**0.039**
Chronic kidney disease	2.44 (1.41–4.23)	**0.001**	2.46 (1.41–4.30)	**0.002**	—	—	2.10 (1.20–3.67)	**0.009**
Coronary artery disease	1.11 (0.66–1.87)	0.694	1.10 (0.65–1.86)	0.722	—	—	1.07 (0.63–1.82)	0.803
Heart failure	2.60 (1.51–4.48)	**<0.001**	2.58 (1.49–4.49)	**0.001**	—	—	2.47 (1.41–4.32)	**0.002**
Anemia	1.65 (1.02–2.68)	**0.042**	1.66 (1.02–2.70)	**0.041**	—	—	1.56 (0.98–2.50)	0.063
Complete atrioventricular block	—	—	1.89 (1.10–3.25)	**0.021**	—	—	1.55 (0.89–2.70)	0.122
LVEF, per 1%	—	—	—	—	1.00 (0.96–1.05)	0.916	—	—
eGFR, per 1 mL/min/1.73 m^2^	—	—	—	—	0.99 (0.97–1.01)	0.423	—	—
Hemoglobin, per g/dL	—	—	—	—	0.95 (0.73–1.22)	0.671	—	—
Admission heart rate, per 10 bpm increase	—	—	—	—	—	—	0.85 (0.72–1.01)	0.060
Admission systolic blood pressure, per 10 mmHg increase	—	—	—	—	—	—	0.91 (0.84–0.99)	**0.024**
Admission lactate, per 1 mmol/L increase	—	—	—	—	—	—	1.37 (1.19–1.58)	**<0.001**

All models included 698 patients and 101 in-hospital deaths. Model 1 included ln(1 + CAR), age, sex, hypertension, diabetes mellitus, chronic kidney disease, coronary artery disease, heart failure, and anemia. Model 2 additionally included the complete atrioventricular block. Model 3 included ln(1 + CAR), age, sex, LVEF, eGFR, and hemoglobin. Model 4 included all variables in Model 2 plus admission heart rate, systolic blood pressure, and lactate. Dashes indicate variables not included. Bold values indicate *p* < 0.05. CAR, C-reactive protein-to-albumin ratio; CI, confidence interval; eGFR, estimated glomerular filtration rate; ln, natural logarithm; LVEF, left ventricular ejection fraction; OR, odds ratio.

**Table 5:** Multivariable logistic regression models for temporary pacemaker requirement.

Variable	Model 1 OR (95% CI)	*p* Value	Model 2 OR (95% CI)	*p* Value
ln(1 + CAR)	2.25 (1.57–3.21)	<0.001	1.99 (1.38–2.86)	**<0.001**
Age, per year	1.02 (1.00–1.03)	0.020	1.01 (1.00–1.03)	**0.047**
Male sex	0.76 (0.54–1.08)	0.127	0.76 (0.53–1.08)	0.127
Hypertension	1.75 (1.21–2.55)	0.003	1.80 (1.22–2.66)	**0.003**
Diabetes mellitus	1.05 (0.71–1.56)	0.804	1.01 (0.67–1.52)	0.959
Chronic kidney disease	1.25 (0.80–1.95)	0.333	1.18 (0.74–1.88)	0.482
Coronary artery disease	1.25 (0.86–1.82)	0.240	1.28 (0.86–1.90)	0.218
Heart failure	1.18 (0.74–1.87)	0.490	1.06 (0.66–1.73)	0.804
Anemia	0.78 (0.54–1.12)	0.174	0.72 (0.49–1.05)	0.088
Complete atrioventricular block	—	—	2.61 (1.45–4.72)	**0.001**
2:1 atrioventricular block	—	—	0.59 (0.31–1.13)	0.112

Model 1 was adjusted for age, sex, hypertension, diabetes mellitus, chronic kidney disease, coronary artery disease, heart failure, anemia, and ln(1 + CAR). Model 2 was additionally adjusted for complete atrioventricular block and 2:1 atrioventricular block. CAR, C-reactive protein-to-albumin ratio; CI, confidence interval; OR, odds ratio.

**Table 6:** Discriminative performance of C-reactive protein-to-albumin ratio, C-reactive protein, albumin, and C-reactive protein-to-albumin ratio-added clinical models.

**Panel A.** ROC analyses of C-reactive protein-to-albumin ratio, C-reactive protein, and albumin					
**Outcome**	**Marker**	**AUC (95% CI)**	**Youden cut-off**	**Sensitivity (%)**	**Specificity (%)**
In-hospital mortality	C-reactive protein-to-albumin ratio	0.699 (0.642–0.750)	0.391	56.4	72.8
In-hospital mortality	C-reactive protein	0.684 (0.629–0.740)	1.406	55.4	73.0
In-hospital mortality	Albumin	0.690 (0.631–0.748)	3.800	84.2	43.6
Temporary pacemaker requirement	C-reactive protein-to-albumin ratio, mg/g	0.630 (0.586–0.671)	0.267	53.2	68.0
Temporary pacemaker requirement	C-reactive protein	0.615 (0.572–0.659)	1.000	51.5	66.9
Temporary pacemaker requirement	Albumin	0.653 (0.610–0.696)	3.660	61.3	64.9
**Panel B.** Incremental discriminatory value of CAR, CRP, albumin, and CRP plus albumin when added to clinical prediction models					
**Model comparison**	**Base model AUC** **(95% CI)**	**Model + marker AUC** **(95% CI)**	**ΔAUC (95% CI)**	***p*** **value**	
In-hospital mortality clinical model vs. +CAR	0.751 (0.701–0.798)	0.782 (0.738–0.828)	0.031 (0.005–0.058)	0.020	
In-hospital mortality clinical model vs. +CRP	0.751 (0.701–0.798)	0.783 (0.735–0.826)	0.032 (0.004–0.061)	0.023	
In-hospital mortality clinical model vs. +albumin	0.751 (0.701–0.798)	0.774 (0.727–0.820)	0.024 (−0.007–0.055)	0.142	
In-hospital mortality clinical model vs. +CRP + albumin	0.751 (0.701–0.798)	0.792 (0.746–0.834)	0.041 (0.008–0.075)	0.014	
Temporary pacemaker clinical model vs. +CAR	0.623 (0.579–0.666)	0.671 (0.629–0.709)	0.048 (0.019–0.074)	<0.001	
Temporary pacemaker clinical model vs. +CRP	0.623 (0.579–0.666)	0.673 (0.631–0.713)	0.050 (0.017–0.081)	0.002	
Temporary pacemaker clinical model vs. +albumin	0.623 (0.579–0.666)	0.699 (0.656–0.739)	0.075 (0.039–0.112)	<0.001	
Temporary pacemaker clinical model vs. +CRP + albumin	0.623 (0.579–0.666)	0.711 (0.670–0.751)	0.088 (0.049–0.126)	<0.001	
Temporary pacemaker clinical + AV block phenotype model vs. +CAR	0.712 (0.669–0.747)	0.730 (0.692–0.768)	0.018 (0.001–0.035)	0.038	
Temporary pacemaker clinical + AV block phenotype model vs. +CRP	0.712 (0.669–0.747)	0.730 (0.691–0.769)	0.018 (−0.000–0.037)	0.055	
Temporary pacemaker clinical + AV block phenotype model vs. +albumin	0.712 (0.669–0.747)	0.749 (0.711–0.788)	0.037 (0.013–0.060)	0.004	
Temporary pacemaker clinical + AV block phenotype model vs. +CRP + albumin	0.712 (0.669–0.747)	0.756 (0.718–0.792)	0.044 (0.019–0.069)	<0.001	

For albumin, values below the Youden-derived cut-off indicated higher risk; for C-reactive protein and C-reactive protein-to-albumin ratio, values above the cut-off indicated higher risk. AUC, area under the receiver operating characteristic curve; AV, atrioventricular; CAR, C-reactive protein-to-albumin ratio; CI, confidence interval; CRP, C-reactive protein; ROC, receiver operating characteristic.

## Data Availability

The data presented in this study are available from the corresponding author upon reasonable request and subject to institutional and ethical approval. The data are not publicly available due to privacy and ethical restrictions related to patient-level clinical data.

## Supplementary Materials

The following supporting information can be downloaded at https://www.mdpi.com/article/10.3390/medicina62101993/s1. Figure S1: Decision-curve analysis of biomarker-augmented clinical models. Table S1: Causes and timing of in-hospital death according to temporary pacemaker requirement. Table S2: Bootstrap internal validation of the mortality models. Table S3: Medication-adjusted sensitivity logistic regression models. Table S4: Adjusted logistic regression models using high C-reactive protein-to-albumin ratio defined as the fourth quartile. Table S5: Internal validation, calibration, and overall performance of biomarker-augmented clinical models.

## Abbreviations

AST	Aspartate aminotransferase
ALT	Alanine aminotransferase
AF	Atrial fibrillation
AV	Atrioventricular
CV	Cardiovascular
CKD	Chronic kidney disease
CAD	Coronary artery disease
CRP	C-reactive protein
CAR	C-reactive protein-to-albumin ratio
eGFR	Estimated glomerular filtration rate
HF	Heart failure
LVEF	Left ventricular ejection fraction
PPI	Permanent pacemaker implantation
ROC	Receiver operating characteristic
TPR	Temporary pacemaker requirement
