# Supplementary material for: Prognostic Value of the C-Reactive Protein-to-Albumin Ratio in Patients Hospitalized with Atrioventricular Block: Association with In-Hospital Mortality and Temporary Pacemaker Requirement
Source: Medicina (Kaunas). 2026 Oct 15;62(10):1993. doi: 10.3390/medicina62101993 (PMC13706709; doi:10.3390/medicina62101993)
Supplement: Supplementary file 1 [file medicina-62-01993-s001.zip › medicina-4564005-supplementary.pdf]

Supplementary Table S1: Causes and timing of in-hospital death according to temporary pacemaker requirement.

Variable	All non-survivors (n = 101)	No temporary PM (n = 56)	Temporary PM (n = 45)
**Primary cause of death, n (%)**			
Cardiogenic shock/progressive heart failure	31 (30.7)	16 (28.6)	15 (33.3)
Hospital-acquired infection/sepsis	22 (21.8)	13 (23.2)	9 (20.0)
Arrhythmic death	14 (13.9)	5 (8.9)	9 (20.0)
Respiratory failure	12 (11.9)	8 (14.3)	4 (8.9)
Multiple organ failure/progressive renal failure	9 (8.9)	5 (8.9)	4 (8.9)
Stroke/intracranial hemorrhage	4 (4.0)	3 (5.4)	1 (2.2)
Procedure-related complication	3 (3.0)	1 (1.8)	2 (4.4)
Other or undetermined	6 (5.9)	5 (8.9)	1 (2.2)
**Timing of death, n (%)**			
≤48 h after admission	24 (23.8)	10 (17.9)	14 (31.1)
>48 h to seven days after admission	38 (37.6)	20 (35.7)	18 (40.0)
>Seven days after admission	39 (38.6)	26 (46.4)	13 (28.9)

Supplementary Table S2: Bootstrap internal validation of the mortality models.

Model	Patients, n	Deaths, n	Apparent AUC	Mean optimism	Optimism-corrected AUC	Optimism-corrected calibration slope	Successful bootstrap replicates
Model 1	698	101	0.782	0.025	0.757	0.885	1000/1000
Model 2	698	101	0.787	0.026	0.761	0.878	1000/1000
Model 3	698	101	0.689	0.090	0.599	0.670	1000/1000
Model 4	698	101	0.844	0.023	0.821	0.875	1000/1000

Internal validation used 1000 bootstrap resamples, with each model refitted in every resample. Model specifications are provided in Table 4. AUC, area under the receiver operating characteristic curve.

Supplementary Table S3: Medication-adjusted sensitivity logistic regression models

Model	Variable	OR (95% CI)	*p* value
Mortality clinical + medication model	ln(1 + CAR)	1.80 (1.28–2.54)	**<0.001**
Mortality clinical + medication model	Any AV nodal/conduction-slowing drug	0.68 (0.41–1.14)	0.144
Mortality + AV block phenotype + medication model	ln(1 + CAR)	2.11 (1.42–3.15)	**<0.001**
Mortality + AV block phenotype + medication model	Any AV nodal/conduction-slowing drug	0.71 (0.42–1.18)	0.187
Mortality + AV block phenotype + medication model	Complete atrioventricular block	1.88 (1.09–3.24)	**0.022**
Temporary PM clinical + medication model	ln(1 + CAR)	1.81 (1.29–2.53)	**<0.001**
Temporary PM clinical + medication model	Any AV nodal/conduction-slowing drug	1.21 (0.86–1.72)	0.278
Temporary PM + AV block phenotype + medication model	ln(1 + CAR)	1.61 (1.16–2.23)	**0.005**
Temporary PM + AV block phenotype + medication model	Any AV nodal/conduction-slowing drug	1.17 (0.81–1.69)	0.402
Temporary PM + AV block phenotype + medication model	Complete atrioventricular block	2.79 (1.55–5.02)	**<0.001**
Temporary PM + AV block phenotype + medication model	2:1 atrioventricular block	0.60 (0.32–1.14)	0.120

Clinical models included ln(1 + CAR), age, sex, hypertension, diabetes mellitus, chronic kidney disease, coronary artery disease, heart failure, and anemia, with additional adjustment for any AV nodal/conduction-slowing drug use. The phenotype-adjusted mortality model additionally included complete atrioventricular block. The phenotype-adjusted temporary PM model additionally included complete and 2:1 atrioventricular block. Temporary pacing was not included as a covariate in the mortality models. Only selected coefficients are shown. Bold values indicate $p < 0.05$.

AV, atrioventricular; CAR, C-reactive protein-to-albumin ratio; CI, confidence interval; OR, odds ratio; PM, pacemaker.

Supplementary Table S4: Adjusted logistic regression models using high C-reactive protein-to-albumin ratio defined as the fourth quartile

Endpoint	Variable	OR (95% CI)	*p* value
In-hospital mortality	CAR Q4 vs. Q1–Q3	2.54 (1.59–4.08)	**<0.001**
In-hospital mortality	Age, per year	1.02 (1.00–1.04)	**0.095**
In-hospital mortality	Male sex	1.25 (0.77–2.03)	0.363
In-hospital mortality	Hypertension	0.82 (0.50–1.34)	0.433
In-hospital mortality	Diabetes mellitus	0.52 (0.28–0.97)	0.039
In-hospital mortality	Chronic kidney disease	2.54 (1.46–4.40)	**<0.001**
In-hospital mortality	Coronary artery disease	1.06 (0.63–1.79)	0.820
In-hospital mortality	Heart failure	2.57 (1.49–4.43)	**<0.001**
In-hospital mortality	Anemia	1.76 (1.09–2.85)	**0.021**
Temporary pacemaker requirement	CAR Q4 vs. Q1–Q3	2.50 (1.71–3.65)	**<0.001**
Temporary pacemaker requirement	Age, per year	1.02 (1.00–1.03)	**0.025**
Temporary pacemaker requirement	Male sex	0.75 (0.53–1.06)	0.105
Temporary pacemaker requirement	Hypertension	1.76 (1.21–2.55)	**0.003**
Temporary pacemaker requirement	Diabetes mellitus	1.06 (0.71–1.56)	0.788
Temporary pacemaker requirement	Chronic kidney disease	1.30 (0.83–2.02)	0.249
Temporary pacemaker requirement	Coronary artery disease	1.23 (0.85–1.79)	0.278
Temporary pacemaker requirement	Heart failure	1.16 (0.73–1.85)	0.527
Temporary pacemaker requirement	Anemia	0.81 (0.56–1.16)	0.247

Models were adjusted for age, sex, hypertension, diabetes mellitus, chronic kidney disease, coronary artery disease, heart failure, anemia, and high C-reactive protein-to-albumin ratio. CAR, C-reactive protein-to-albumin ratio; CI, confidence interval; OR, odds ratio.

Supplementary Table S5: Internal validation, calibration, and overall performance of biomarker-augmented clinical models

Model set	Biomarker specification	Apparent AUC	Optimism-corrected AUC	Apparent Brier score	Optimism-corrected Brier score	Optimism-corrected calibration intercept	Optimism-corrected calibration slope	Successful bootstrap replicates
In-hospital mortality clinical model	Base model	0.751	0.725	0.112	0.116	−0.179	0.886	1000/1000
In-hospital mortality clinical model	+CAR	0.782	0.759	0.109	0.114	−0.168	0.890	1000/1000
In-hospital mortality clinical model	+CRP	0.783	0.759	0.109	0.113	−0.161	0.893	1000/1000
In-hospital mortality clinical model	+Albumin	0.774	0.750	0.109	0.114	−0.161	0.885	1000/1000
In-hospital mortality clinical model	+CRP + albumin	0.792	0.767	0.107	0.113	−0.167	0.884	1000/1000
Temporary pacemaker clinical model	Base model	0.623	0.595	0.214	0.220	−0.131	0.796	1000/1000
Temporary pacemaker clinical model	+CAR	0.671	0.646	0.208	0.214	−0.103	0.837	1000/1000
Temporary pacemaker clinical model	+CRP	0.673	0.647	0.207	0.214	−0.101	0.841	1000/1000
Temporary pacemaker clinical model	+Albumin	0.699	0.678	0.202	0.208	−0.073	0.878	1000/1000
Temporary pacemaker	+CRP + albumin	0.711	0.690	0.199	0.206	−0.071	0.879	1000/1000

Model set	Biomarker specification	Apparent AUC	Optimism-corrected AUC	Apparent Brier score	Optimism-corrected Brier score	Optimism-corrected calibration intercept	Optimism-corrected calibration slope	Successful bootstrap replicates
clinical model								
Temporary pacemaker clinical + AV block phenotype model	Base model	0.712	0.692	0.197	0.203	−0.059	0.893	1000/1000
Temporary pacemaker clinical + AV block phenotype model	+CAR	0.730	0.710	0.192	0.200	−0.068	0.883	1000/1000
Temporary pacemaker clinical + AV block phenotype model	+CRP	0.730	0.710	0.192	0.199	−0.058	0.893	1000/1000
Temporary pacemaker clinical + AV block phenotype model	+Albumin	0.749	0.731	0.186	0.194	−0.054	0.902	1000/1000
Temporary pacemaker clinical + AV block phenotype model	+CRP + albumin	0.756	0.736	0.185	0.193	−0.064	0.889	1000/1000

AUC, area under the receiver operating characteristic curve; AV, atrioventricular; CAR, C-reactive protein-to-albumin ratio; CRP, C-reactive protein.

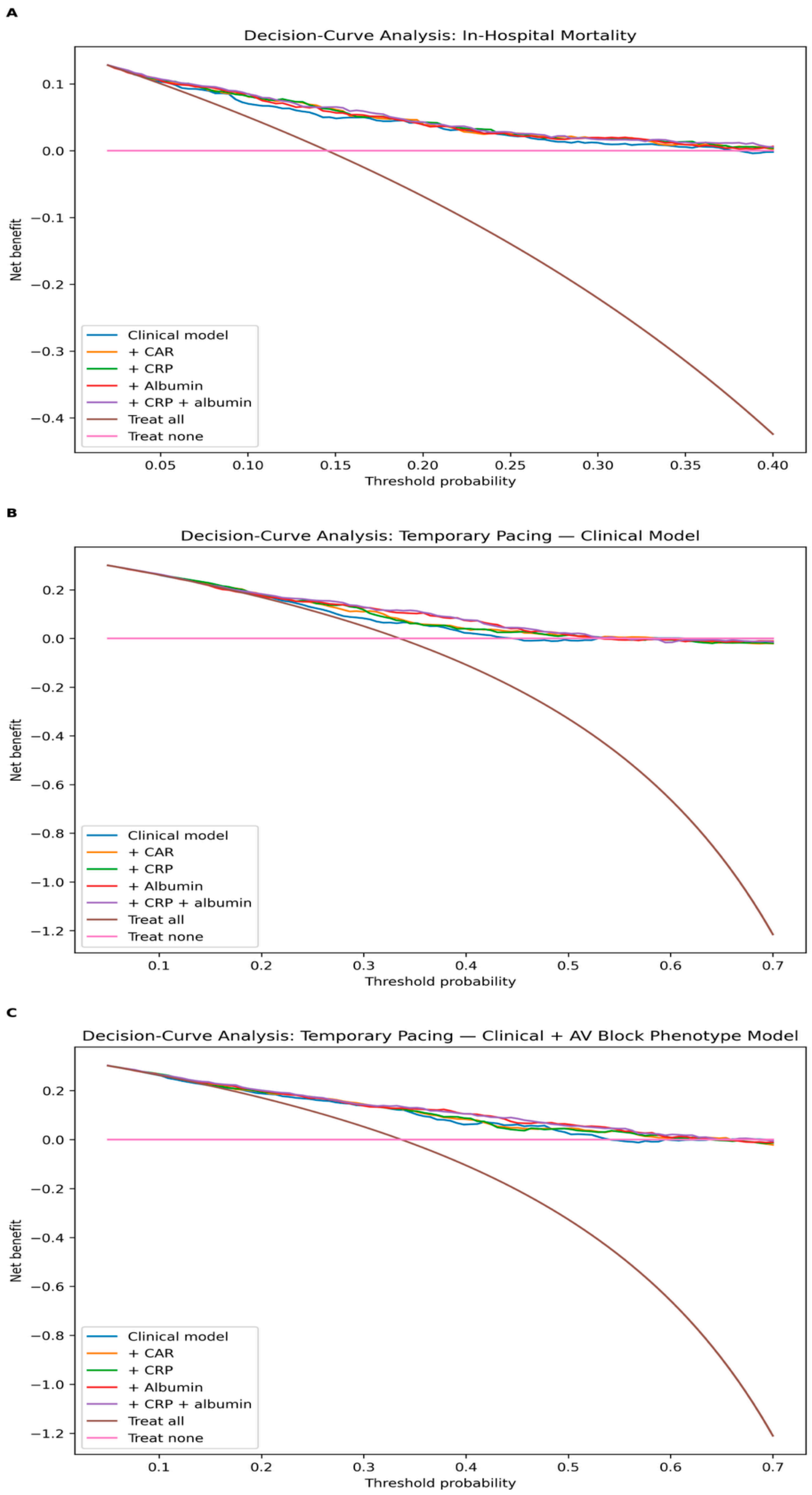

Supplementary Figure S1: Decision-curve analysis of biomarker-augmented clinical models. (A) In-hospital mortality clinical models; (B) temporary pacemaker clinical models; and (C) temporary pacemaker clinical models additionally incorporating AV block

phenotype. Net benefit was evaluated across clinically relevant threshold probabilities for the base model and models supplemented with CAR, CRP, albumin, or CRP plus albumin. Differences in net benefit were threshold-dependent, and CAR did not demonstrate consistent superiority over the alternative biomarker models.

AV, atrioventricular; CAR, C-reactive protein-to-albumin ratio; CRP, C-reactive protein.
